# A New Isoflavonoid from Seeds of *Lepidium sativum* L. and Its Protective Effect on Hepatotoxicity Induced by Paracetamol in Male Rats

**DOI:** 10.3390/molecules191015440

**Published:** 2014-09-26

**Authors:** Mohamed Sakran, Yasser Selim, Nahla Zidan

**Affiliations:** 1 Department of Chemistry, Faculty of Science, Tanta University, Tanta 31527, Egypt; E-Mail: msakran2011@gmail.com; 2 Department of Chemistry and Biochemistry, Faculty of Science, Tabuk University, Tabuk P.O. Box 699, Tabuk 71491, Saudi Arabia; 3 Faculty of Specific Education, Zagazig University, Zagazig 44519, Egypt; 4 Department of Basic and Clinical Medical Science, Faculty of Dentistry, P. Qaseem University, Qaseem 51411, Saudi Arabia; 5 Department of Home Economics, Faculty of Specific Education, Kafr ElSheikh University, Kafr ElSheikh 33516, Egypt; E-Mail: nahlasalah2008@yahoo.com

**Keywords:** *Lepidium sativum*, isoflavonoid, hepatotoxicity

## Abstract

A new isoflavonoid, 5,6-dimethoxy-2',3'-methylenedioxy-7-C-β-d-gluco-pyranosyl isoflavone was isolated from the seeds of *Lepidium sativum* L. along with two known isoflavonoids, 7-hydroxy-4',5,6-trimethoxyisoflavone and 7-hydroxy-5,6-dimethoxy-2',3'-methylenedioxyisoflavone. The structures of all compounds were elucidated with NMR spectrometry. Compounds **1**, **2** and the new isoflavonoid **3** were evaluated for their ability to reduce the hepatotoxicity induced by paracetamol in male rats by reducing the damage and toxicity effects on liver cells with a significant improvement of total antioxidant capacity, normalizing the levels of liver enzymes GSH, SOD, GPX, CAT and GST compared to control group.

## 1. Introduction

*L. sativum* (family Cruciferae) grows in Egypt by three species: *L.* *latifolium*, *L. sativum* and *L. aucheri*, of which the most common one is *L. sativum* [1]. It was assumed that its seeds can be a functional food. Eaten plants and the seed oils are used in treating dysentery, diarrhea [2] and migraine [3]. *L. sativum* is popular medical treatments used in the Kingdom of Saudi Arabia, Sudan and some other Arab countries and mediator well for healing broken bones in the human skeleton. He noted a number of recent studies of the traditional uses of *L. sativum* seed extract in control of many of the clinical problems. It was found that oral administration of aqueous extract of *L. sativum* caused a significant drop in blood pressure [4]. Some of these activities are responsible for the ability of *L. sativum* to protect DNA from damage caused by free radical and protect liver cells against various toxins [5]. There are some reports in the *in vitro* and *in vivo* experiments Antimutagenic activities, anti-cancer and anticholestatic using *L. sativum* [5]. The chemical analyses showed the identification of 6'-β-rhamnopyranosil-oleoside, 6'-β-glucopyranosil-oleoside, Luteolin and of organic polymeric component, known as polymerin [6]. In this paper, we report the isolation of a new one and two of the known compounds and *in vivo* study of the effects of the new complex liver **3** and two other compounds 1 and 2 against liver damage caused by paracetamol.

## 2. Results and Discussion

### 2.1. Chemistry

**Figure 1:**
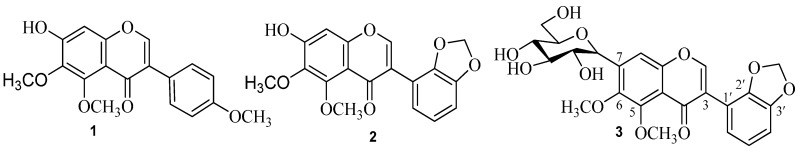
Structures of the isolated compounds.

Seeds of *L. sativum* were divided into petroleum ether, ethyl acetate and methanol fractions. The methanolic fraction was purified by repeated silica gel column chromatography to give compounds **1**–**3** (Figure 1). The known compounds **1** and **2** were identified as 7-hydroxy-4',5,6-trimethoxyisoflavone [7] and 7-hydroxy-5,6-dimethoxy-2',3'-methylenedioxyisoflavone [8], respectively, by comparison of their NMR, MS and physical data with those described in the corresponding literatures. This is the first report of natural isolation of compound **3**. In our initial biological study, as shown in Table 2 and Table 3, compound **3** showed higher ability to reduce the hepatotoxicity induced by paracetamol than compounds **1** and **2**. This effect could help explain the use of *L.sativum* in traditional medicine.

**Figure 2:**
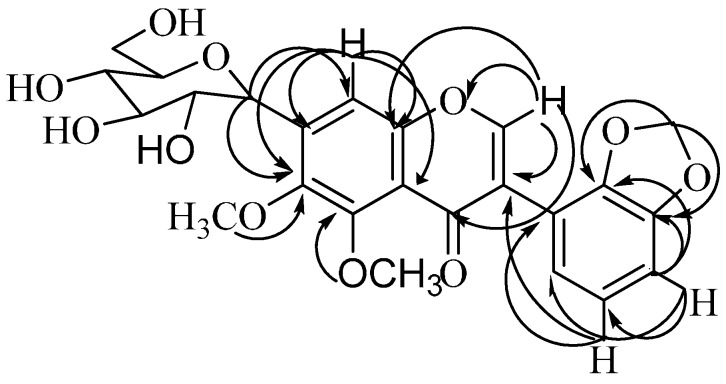
HMBC correlation of compound **3**.

Compound **3** was isolated as a white solid, which was further crystallized from acetone. It showed a molecular-ion peak at *m*/*z* 489.1317 corresponding to the molecular formula C_24_H_25_O_11_. A retro-Diels-Alder reaction (RDA) is the main fragmentation pattern observed in the ESI spectra, resulting in a diagnostic peak at *m*/*z* 149 corresponding to the (OCH_2_O) C_6_H_3_–C≡O^+^ fragment, consistent with a 2',3'-methylenedioxy substitution pattern on the B-ring [9]. The UV spectrum exhibited absorption maxima at 250 and 314 nm, which are characteristic of an isoflavonoid skeleton [10]. Its IR spectrum showed a chelated carbon at 1700 cm^−1^ (*γ-*pyrone nucleus) along with other absorption bands at 1600 and 859 cm^−1^, a characteristic of an aromatic nucleus. The FTIR spectrum of compound **3** shows a broad absorption band around 3489 cm^−1^ that indicated the presence of hydroxyl groups as expected. The band at 2954 cm^−1^ is attributed to the CH moieties in the molecule [11]. In the ^1^H-NMR spectrum of **3** (Table 1), the characteristic resonance for H-2 of an isoflavonoid was observed at δ 8.5 [12]. This assignment was confirmed by long-range connectivities to the quaternary carbons at δ 180.5 (C-4), 150.3 (C-9), 121.7 (C-3), and 120.8 (C-1') in the HMBC spectrum. The ^1^H-NMR spectrum of **3** (Table 1) showed the presence of two methoxy groups [δ 3.79 (3H, s) and 3.83 (3H, s)] and one methylenedioxy group [δ 6.07 (2H, s)]. In addition, protons at δ 6.76 (1H, d, *J* = 1.95 Hz, H-4'), 6.98 (1H, d, *J* = 2.0 Hz, H-5') and 7.15 (1H, d, *J* = 1.95Hz, H-6')] [9], suggested that C-2' and C-3' were substituted. Moreover a singlet at δ 6.87, corresponding to H-8, showed HMBC correlations with quaternary carbons at δ 150.3 (C-9), 144.2 (C-6),131.5 (C-7) and 116.3 (C-10) [13], as well as H-2 (δ 8.5) showed long-range HMBC connectivities to the carbonyl carbon (δ 180.5, C-4), two quaternary carbons (δ 121.7, C-3 and 122.3, C-6'), and the quaternary oxygenated carbon C-9 (δ 150.3). The ^13^C-NMR spectrum (Table 1), revealed the presence of two methoxy methyl groups, two methylene groups, ten methane carbons and ten quaternary carbon atoms. In addition, the ^13^C-NMR spectrum showed two methoxy carbon signals resonating at lower magnetic field δ 60.6 (H-6) and 61.8 (H-5). This suggested that both positions *ortho* to the methoxy group were substituted [8,9,13]. The carbon shifts of OCH_3_ substituent usually occur between δ 55.0 and 56.5, but in some cases they are observed further downfield between δ 60.6 and 61.8 [8]. This deshielding effect is seen only when the OCH_3_ is di*-ortho-*substituted. From the ^1^H spectrum, two one proton doublets resonated at δ 4.82 and 3.81 and were assigned to H-1'' (*J* = 8.9, anomeric) and H-6'' (*J* = 12.3). Two one proton doublets of doublets at δ 3.75 (*J* = 8.9, 9.2) and 3.54 (*J* = 6.2, 12.5) were attributed to H-2'' and H-6''. Multiplet at δ 3.37 (*J* = 8.9), 3.41 (*J* = 8.9) and 2.94 (*J* = 6.2, 8.8) corresponding to H-3'', H-4'' and H-5'' were also seen. The position of the sugar was confirmed by HMBC long-range correlation in which the anomeric H-1'' showed long-range coupling with the signals at 144.2 (C-6), 131.3 (C-7), and 107.8 (C-8) ppm, suggesting the sugar position to be at C-7 of aromatic ring A (Figure 2). Please confirm the highlights.

The appearance of an anomeric carbon (73.7) and proton (4.82) related to those of aromatic C-glycoside data and the anomeric proton correlation with C-6, C-7, and C-8 in the HMBC experiment elucidated its C-glycosidic nature which was confirmed by its resistance to acidic hydrolysis [14]. All correlations in the HMBC spectrum (Figure 2) were in complete agreement with the proposed structure. From the above data, compound **3** could thus be identified as 5,6-dimethoxy-2',3-methylenedioxy-7-C-β-d-glucopyranosylisoflavone.

**Table 1:** NMR spectroscopic data of the isolated compounds.

Position	1		2		3	
	^1^H	^13^C	^1^H	^13^C	^1^H	^13^C
2	8.68s	153.1	8.51s	153.6	8.5s	153.2
3	-	124.7	-	120.9	-	121.7
4	-	174.5	-	180.6	-	180.5
5	-	153.2	-	153.6	-	157.2
6	-	137.2	-	137.5	-	144.2
7	-	157.3	-	157.1	-	131.3
8	6.14s	96.1	6.15s	94.7	6.78s	107.8
9	-	151.3	-	151.9	-	150.3
10	-	113.2	-	113.9	-	116.3
1'	-	124.8	-	120.9	-	120.8
2'	7.52 (d2.0)	130.1	-	149.1	-	147.8
3'	6.94 (d8.0)	114.7	-	148.7	-	148.2
4'	-	159.3	6.75 (d1.99)	114.3	6.76 (d1.95)	114.5
5'	6.96 (d2,4)	114.5	7.18 (d2.0)	121.5	6.98 (d2.0)	121.3
6'	7.52 (d2.9)	130.6	6.85 (d1.95)	122.6	7.15 (d1.95)	122.3
1''	-	-	-	-	4.82 (d,8.9)	73.7
2''	-	-	-	-	3.75 (dd8.9,9.2)	84.2
3''	-	-	-	-	3.37 (t8,9)	72.2
4''	-	-	-	-	3.41 (t8.9)	71.3
5''	-	-	-	-	2.94 (ddd6.2, 8.8)	84.6
6''	-	-	-	-	3.81 (d12.3) 3.54 (dd6.2, 12.5)	62.2
OCH_3_-5	3.83s	62.2	3.84s	61.3	3.79s	61.8
OCH_3_-6	3.83s	61.1	3.85s	62.2	3.83s	60.6
OCH_3_-4'	3.86s	55.5	-	-		-
OCH_2_O-3',4'	-	-	6.15s	101.3	6.07s	101.6
OH-7	5.33s	-	5.35s	-	-	-

### 2.2. Biology Assays

#### 2.2.1. Effect of Compounds **1**, **2** and **3** on Liver Function

The data summarized in Table 2 indicated that AST, ALT, ALP, GGT activities were significantly increased (*p* < 0.05) in rats that received paracetamol (group 2) or rats that received paracetemol + compound **1** (group 3) or rats that received paracetemol + compound **2** (group 4) when compared with the control group (group 1). However, no significant differences of these parameters were seen when the compound **3** treated group (group 5) is compared to the control group. There is a also a significant decrease (*p* < 0.05) in AST, ALT, ALP, GGT activities in rats that received paracetemol + compound **1** (group 3) or rats the received paracetemol + compound **2** (group 4) or rats that received paracetemol + compound **3** (group 5) when compared with the paracetemol group (group 2). In the assessment of liver injury by paracetemol, the analysis of levels of enzymes such as AST and ALT is widely used. Necrosis or membrane damage releases the enzymes into circulation and hence they can be measured in the serum. High levels of AST indicate liver damage. ALT is more specific to the liver, and is thus a better parameter for detecting liver injury. Elevated levels of serum enzymes are indicative of cellular leakage and loss of functional integrity of the cell membranes in the liver. Serum ALP and bilirubin levels, on the other hand, are related to the function of hepatic cells. Administration of paracetemol caused a significant (*p* < 0.001) elevation of the levels of enzymes such as AST, ALT, ALP and GGT [15]. In agreement with our results mean serum AST, ALT, ALP levels and bilirubin concentration were significantly increased in the CCl_4_ induced hepatotoxicity group of rats compared to the control (*p* < 0.05). However significant reduction in these parameters were found in groups treated with *Lepidium sativum* [7].

**Table 2:** Levels of serum ALT, AST, ALP and GGT of normal and experimental groups.

Group	ALT(U/L)	AST(U/L)	ALP(U/L)	GGT(U/L)
Control Group 1	40 ± 1.5	43 ± 2.1	33 ± 2.4	60 ± 1.4
Group2: Paracetamol	70 ± 1.6 *	80 ± 2.5 *	71 ± 2.2 *	117 ± 2.5 *
Group 3: Paracetamol + compound 1	60 ± 1.45 *#	60 ± 2.2 *#	53 ± 1.9 *#	91 ± 2.4 *#
Group 4: Paracetamol + compound **2**	61 ± 1.33 *#	62.2 ± 2.3 *#	54 ± 1.8 *#	95 ± 2.7 *#
Group 5: Paracetamol + compound **3**	50 ± 1.2 #	53.5 ± 2.1 #	39 ± 2.1 #	72 ± 1.6 #

Values are expressed as means ± SE; n = 10 for each treatment group; Significant difference from the control group at * *p* ˂ 0.05; Significant difference from paracetemol group at # *p* ˂ 0.05.

#### 2.2.2. Effect of Compounds **1**, **2** and **3** on Oxidative Stress Parameters

Table 3 showed that plasma total antioxidant capacity and liver SOD, GPx, CAT, GST and GSH were significantly decreased (*p* < 0*.05*) in Paracetamol group (Group 2) compared to control group (group 1). However the concentrations and activities of total antioxidant enzymes GST, SOD and CAT were significantly increased when compound **1**, **2** or **3** was administered (group 3, 4 and 5) while animals treated with Paracetamol had a significant increase (*p* < 0.05) in the level of MDA concentration compared to control group as well as treatment with compound **1**, **2** or **3** (groups 3, 4 and 5) significantly overcomes the effect of Paracetamol on hepatic lipid peroxidation by lowering hepatic MDA. lipid peroxidation in the Paracetemol group reveals hepatic damage due to paracetemol ,which was supported by increasing the activities of ALT, AST, ALP and GGT. Compounds 1, 2 and 3 decreases the peroxidation of lipids and SOD activity indicates hepatocellular damage because it scavenges the superoxide anion to form hydrogen peroxide, thus decreases the toxic effect caused by this radical [15]. In agreement with our results *Lepidium sativum* seed extract protects the liver from damage by CCl_4_ and this was supported by histology and biochemical parametes of liver injury. The hepatoprotective action of the plant may be due to the ability of the plant to overcome lipid peroxidation in the liver. *Lepidium sativum* inhibits free radicals mediated damage and so protects from liver injury [7]. The hepatoprotective effect of *Lepidium sativum* is due to flavonoids, triterpens and tannin as antioxidant agents that decrease free radicals formation [16,17,18]. On the basis of results obtained it can be concluded that the methanolic extract of *Lepidium sativum* seeds seems to possess hepatoprotective effect in rats.

**Table 3:** Changes in the levels of plasma total antioxidant capacity (mM/L) and liver MDA, SOD, GPx, CAT, GST and GSH of male albino rats in normal and experimental rats.

Group	TAC (mM/L)	MDA nmole/g tissue	SOD units/g tissue	GPx units/g tissue	CAT mole/min/g tissue	GSH μmol/ mg protein	GST units/g tissue
Group 1: Control	1.2 ± 0.04	160 ± 3.23	60 ± 1.25	150 ± 1.5	0.8 ± 0.02	60 ± 1.3	2.2 ± 0.11
Group 2: Paracetamol	0.52 ± 0.02 *	547 ± 9.46 *	24 ± 2.4 *	112 ± 1.2 *	0.42 ± 0.01 *	35 ± 0.85 *	1.1 ± 0.01 *
Group 3: Paracetamol + compound 1	0.61 ± 0.03 *#	320 ± 8.5 *#	30 ± 2.1 *#	118 ± 1.5 *#	0.56 ± 0.01 *#	40 ± 0.65 *#	1.34 ± 0.01 *#
Group 4: Paracetamol + compound 2	0.65 ± 0.03 *#	323 ± 9.11 *#	33 ± 2.2 *#	115 ± 1.4 *#	0.55 ± 0.01 *#	42 ± 0.54 *#	1.37 ± 0.01 *#
Group 5: Paracetamol + compound 3	0.73 ± 0.02 #	211 ± 5.22 #	36 ± 1.5 #	120 ± 6.5 #	0.621 ± 0.01 #	45.5 ± 1.2 #	1.50 ± 0.8 #

Significant difference from the control group at * *p* ˂ 0.05; Values are expressed as means ± SE; n = 10 for each treatment group; Significant difference from paracetemol group at # *p* ˂ 0.05.

#### 2.2.3. Histopathology

**Figure 3:**
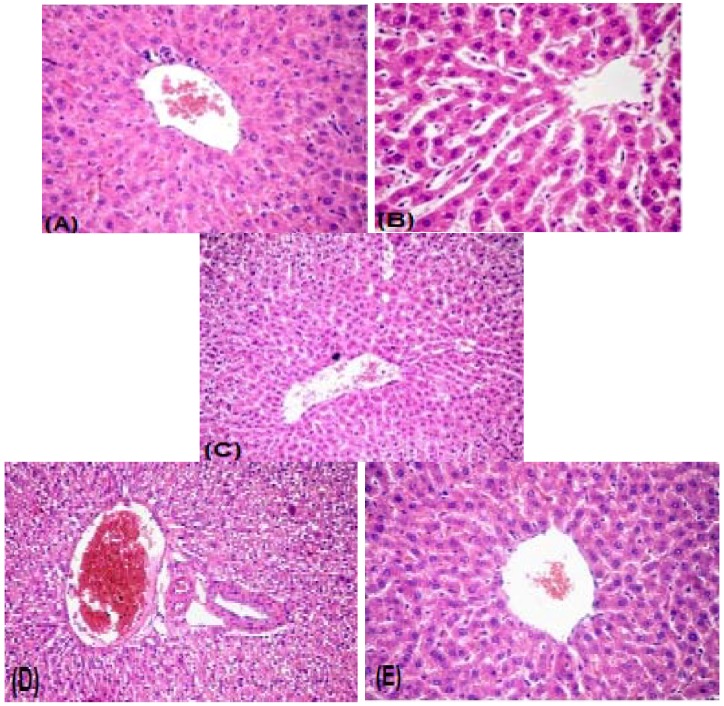
(**A**) normal liver section of rats; (**B**) liver sections in the paracetemol treated group exhibited cirrhosis, and macro nodular structures surrounded by fibrous tissue; (**C**) rats treated with paracetamol plus compound **1**; (**D**) rats treated with paracetamol plus compound **2**; (**E**) rats treated with paracetamol + compound **3**. H&E stain (40×).

Histopathological examination of the liver of control group revealed normal hepatic architecture, cellular and nuclear configurations (Figure 3A). Histopathological alterations observed in the paracetemol-treated group included sinusoidal dilatation and atrophy of hepatocytes (Figure 3B). The livers of rats treated with paracetemol + compound **1** showed no areas of necrosis and they regained their normal structure (Figure 3C). Liver sections of rats treated with paracetemol + compound **2** showed moderate dilatation of portal blood vessels and vacuolar degeneration of hepatocytes (Figure 3D). Livers of rats treated with paracetemol + compound **3** appeared more or less similar to the control sections (Figure 3E).

## 3. Experimental Section

### 3.1. General Information

Optical rotations were measured in MeOH or H_2_O on a Perkin-Elmer241 polarimeter (Manchester, UK) equipped with a sodium lamp (589 nm) and a 1 dm microcell. The melting points were determined using a Digital Melting Point Apparatus (model IA 8103, Electro Thermal Engineering Ltd., Soutthend-on-Sea, UK). The UV spectra were recorded with a Perkin-Elmer Lambda 2UV/VIS spectrophotometer. IR spectra (KBr) were recorded on a Perkin-Elmer 1650 FT-IR spectrometer. ^1^H- and ^13^C-NMR spectra were recorded in CDCl_3_ on a Bruker Avance DRX-500 spectrometer (Oxford, UK) ^1^H at 500 MHz and ^13^C at 125 MHz), and 2D-NMR experiments were performed using Bruker’s standard programs [19]. ESI and high resolution mass spectra were recorded using a Finnigan MAT 90 instrument (Manchester, UK) and a VG Auto Spec-3000 spectrometer (Manchester, UK), respectively. TLC was carried out on precoated silica gel 60 F_254_ (Merck, Munich, Germany), and spots were visualized by heating after spraying with 50% H_2_SO_4_. Column chromatography was carried out on silica gel 60 (63–200 μm, Merck).

### 3.2. Plant Material

*Lepidium sativum* L. seeds were collected from a local market in Egypt (May 2012), authenticated by Prof. Dr. F. Gamal, Prof. of the Aromatic and Medicinal Plants, Botany Department, Faculty of Science, Zagazig University (Zagazig, Egypt). The voucher specimen of *L. sativum* is number L2620 and was kept in a dark and dry container.

### 3.3. Plant Extract

The dried, powdered roots (750 g) were extracted successively with petroleum ether, EtOAc, and MeOH (3 L each) for 96 h to yield after solvent evaporation the corresponding petroleum ether (4.1 g), EtOAc (3.8 g), and MeOH (25.4 g) extracts. The methanol crude extract was chromatographed over silica gel (800 g) and eluted with mixtures of 100% CHCl_3_-100% MeOH as eluents to give three fractions F1 to F3 (9:1, 8.5:1.5, 8:1 v/v). Fraction F2 (7.4 g) was rechromatographed on silica gel with CHCl_3_/MeOH mixtures (9:1, 8.5:1.5, 8:1 v/v) to yield compounds **1** (60 mg), **2** (95 mg) and **3** (126 mg). See Figure 1.

### 3.4. 7-Hydroxy-4',5,6-trimethoxyisoflavone (**1**)

White amorphous solid; m.p. 265–267 °C; UV (MeOH) 275, 331 nm; IR (KBr) ν_max_ 3440, 2955, 1658, 1565, 1430, 1381, 1291, 1153, 1061, 1036, 841 cm^−1^; ^1^H- and ^13^C-NMR (Table 1). The melting point was identical to that of an authentic sample and the value reported for this compound [6]; HRESIMS: 329. 0945 ([M + H]^+^, C_18_H_17_O_6_; calc.329.3160).

### 3.5. 7-Hydroxy-5,6-dimethoxy-2',3'-methylenedioxyisoflavone (**2**)

White amorphous solid; m.p. 270–271 °C; UV (MeOH) λ_max_ 259, 317 nm; IR (KBr) ν_max_ 3451, 2958, 1648, 1622, 1571, 1463, 1373, 1297, 1229, 1147, 1071, 1042 ,817 cm^−1^; ^1^H- and ^13^C-NMR (Table 1). The melting point was identical to that of an authentic sample and the value reported for this compound [7]; HRESIMS: 343.0738 ([M + H]^+^, C_18_H_15_O_7_; calc.343.2995).

### 3.6. 5,6-Dimethoxy-2',3'-methylenedioxy-7-C-β-d-glucopyranosylisoflavone (**3**)

White amorphous solid (purity of compound > 98%); m.p. 277–279 °C; UV (MeOH) λ_max_ nm 250, 314. IR (KBr) ν_max_ cm^−1^: 3,489, 2,954, 1,700, 1,600, 1,591, 1,343, 1,081, 901, 859. ^1^H- and ^13^C-NMR see Table 1. ESIMS: 489 (12*,* M+H), 461 (14), 451 (31), 427 (11), 149 (94), 261 (2), 203 (2), 136 (3). HRESIMS: 489.1317 ([M + H]^+^, C_24_H_25_O_11_; calc.489.4405).

### 3.7. Animals

Adult Sprague Dawley male rats (albino) weighing around 180 to 200 g were purchased from the breeding unit of the Egyptian Organization for Biological Products and Vaccines (Abbassia, Cairo, Egypt). The animals were housed in steel mesh cages and maintained for a one week acclimatization period on commercial standard and pellet diet and drinking water *ad libitum*. The housing cycle was 12:12 h light-dark cycle under controlled temperature (20–22 °C). The animal use protocol had been approved by the Institutional Animals Ethics Committee (IAEC) of Tanta University.

### 3.8. Animal Treatments

The rats were fasted overnight (16–18 h) prior to administration of an oral dose (100 mg/kg) of paracetemol once a day for eight weeks dissolved in sterile normal saline warmed to 40 °C. The rats were divided into five experimental groups of 10 animals each. Group 1 (control group); rats were orally administered with normal saline (1 mL/kg) for eight weeks; Group 2 (paracetemol group); rats were orally administered with paracetamol (100 mg/kg b.wt.) for 8 weeks. Group 3; paracetemol (100 mg/kg b.wt.) + compound **1** treated group (100 mg/kg b.wt/week). Group 4; paracetemol + compound **2** treated group (100 mg/kg b.wt/week) Group 3; paracetemol + compound **3** treated group (100 mg/kg b.wt/week). For co-administration of paracetemol and *L. sativum*, compounds **1**, **2** and **3** were gaved at a dose of 100 mg/kg immediately after oral administration of paracetemol for eight weeks. We have selected the random dose of paracetemol from the literature. Regarding the efficacy of *L. sativum* we have chosen a minimal effective dose of 100 mg/kg b. wt. which shows a great response against paracetemol intoxication. Though we have tested up to 2000 mg/kg b.wt dose and observed no signs of mortality, toxicity and gross behavioral changes [15].

### 3.9. Sample Preparation

Animals were fasted overnight and for clinical chemistry blood samples were obtained from the orbital sinus using capillary tubes (with and without heparin as per requirement) under mild ether anesthesia. Blood for hematology studies was collected into tubes containing ethylenediaminetetra- acetic acid (EDTA) as an anti-coagulant. After animals were sacrificed, the liver was instantly removed, washed three times in ice cold saline and blotted on ash free filter paper, then used for preparation of tissue homogenates for estimation of tissue malondialdehyde (MDA) and glutathione (GSH) levels and the activity of superoxide dismutase (SOD), glutathione peroxidase (GPx), glutathione *S*-transferase (GST) and catalase (CAT) enzyme levels.

### 3.10. Biochemical Analysis

Serum was used to estimate the following liver enzymes: alanine transaminase (ALT) and aspartate transaminase (AST) [20], alkaline phosphatase (ALP) [21], γ-glutamyl transferase (GGT) [22], Total antioxidant capacity was measured in plasma [23]. Hepatic malondialdehyde (MDA) level was estimated [24]. The activity of glutathione* S*-transferase (GST) was measured [25]. The catalase activity in tissue supernatant was measured [26], as were superoxide dismutase (SOD) [27], reduced glutathione [28] and glutathione peroxidase (GPx) levels [29].

### 3.11. Preparation of Tissue Homogenates

Tissue homogenates were prepared as reported by Sakran* et al.* [15]. Briefly, specimens were separated into two parts. Each piece was weighed and homogenized separately with a Potter Elvenhjem tissue homogenizer. The crude tissue homogenate was centrifuged at 11,739 rpm, for 15 min in a cold centrifuge and the resultant supernatant was used for the different estimations [30]. Briefly, liver were immediately excised, washed using chilled saline solution, blotted, weighed and processed for biochemical studies. A small piece of each was immediately fixed in 10% formalin. These formalin-fixed tissues were embedded in paraffin, sectioned (5 μm), stained with hematoxylin and eosin (H&E), and examined under a light microscope for histopathological assessment.

### 3.12. Statistical Analysis

Statistical analysis using SPSS software version 16 [31] was performed using one-way analysis of variance (ANOVA) to assess significant differences among different groups [32]. The results are considered to be significant when *p* *˂* 0.05.

## 4. Conclusions

A new isoflavone *C*-glycoside was isolated from the seeds of *L. sativum* (Asali). Based on spectroscopic techniques and chemical evidence, its structure was elucidated as 5,6-dimethoxy-2',3'-methylenedioxy-7-C-β-d-glucopyranosylisoflavone (**3**) The present study indicated that compound **3** could improve the liver functions, the lipid profile in serum and decrease the generation of free radicals by inducing the antioxidant defense mechanism. New isoflavonoid **3** could be used as a potential antioxidant against paracetamol intoxication with its antioxidant properties and could restore the normal liver functions.
